# Fucoidan and microtopography on polyvinyl alcohol hydrogels guided axons and enhanced neuritogenesis of pheochromocytoma 12 (PC12) cells

**DOI:** 10.1088/1748-605X/ace5fe

**Published:** 2023-07-24

**Authors:** Yuan Yao, Fan Feng, Dency David, Evelyn K F Yim

**Affiliations:** 1 Department of Chemical Engineering, University of Waterloo, 200 University Avenue West, Waterloo, ON N2L 3G1, Canada; 2 Waterloo Institute for Nanotechnology, University of Waterloo, 200 University Avenue West, Waterloo, ON N2L 3G1, Canada; 3 Center for Biotechnology and Bioengineering, University of Waterloo, 200 University Avenue West, Waterloo, ON N2L 3G1, Canada

**Keywords:** topography, axon guidance, fucoidan, neuritogenesis, artificial nerve graft, polyvinyl alcohol

## Abstract

Artificial nerve grafts that support axon growth hold promises in promoting nerve regeneration and function recovery. However, current artificial nerve grafts are insufficient to regenerate axons across long nerve gaps. Specific biochemical and biophysical cues are required to be incorporated to artificial nerve grafts to promote neural cell adhesion and guide neurite outgrowth. Polyvinyl alcohol (PVA) nerve conduits have been clinically approved, but the applicability of PVA nerve conduits is limited to short injuries due to low cell binding. In this study, we explored the incorporation of biochemical cues and topographical cues for promoting neuritogenesis and axon guidance. PVA was conjugated with extracellular matrix proteins and fucoidan, a bioactive sulfated polysaccharide, to improve cell adhesion. Micro-sized topographies, including 1.8 μm convex lenses, 2 μm gratings, and 10 μm gratings were successfully fabricated on PVA by nanofabrication, and the synergistic effects of topography and biochemical molecules on pheochromocytoma 12 (PC12) neuritogenesis and neurite alignment were studied. Conjugated fucoidan promoted the percentage of PC12 with neurite outgrowth from 0% to 2.8% and further increased to 5% by presenting laminin on the surface. Additionally, fucoidan was able to bind nerve growth factor (NGF) on the surface and allow for PC12 to extend neurites in NGF-free media. The incorporation of 2 μm gratings could double the percentage of PC12 with neurite outgrowth and neurite length, and guided the neurites to extend along the grating axis. The work presents a promising strategy to enhance neurite formation and axon guidance, presenting significant value in promoting nerve regeneration.

## 1. Introduction

Peripheral nerve injuries are common clinical problems worldwide. Multiple factors, such as physical trauma, disease, and aging, can lead to peripheral nerve injuries, which result in pain, sensory loss, reduced motor function, and disability. Peripheral nervous system inherently has axonal regeneration capability and can repair a short damaged nerve gap following injury. However, regenerating axons across a longer nerve gap is hindered by several challenges, such as apoptosis of neurons, inflammation, and scar tissue formation [1, 2]. Nerve grafting is commonly required to bridge nerve gaps that are longer than 2–3 cm. The clinical gold standards for nerve grafting are autografts or allografts, which, however, were limited with the shortage of donor grafts, infection risk, and donor-site morbidity [3]. Alternatives using artificial nerve guidance conduits that support neuronal growth become viable options to reconnect injured nerves and promote nerve regeneration [3, 4]. Artificial nerve grafts made of collagen and synthetic materials, such as polyglycolic acid and polyvinyl alcohol (PVA) have been approved by Food and Drug Administration (FDA) and are clinically available for bridging short nerve defects [5]. However, these grafts fail to achieve axon growth across long nerve gaps. Therefore, modification strategies for artificial nerve grafts to provide axon guidance and enhance neurite outgrowth are in need.

Neurite outgrowth and axon guidance are crucial for nerve regeneration and function recovery. The activities and functions of neurons are largely dependent on the biochemical and biophysical cues from their surrounding extracellular environment [6, 7]. Numerous studies have demonstrated that topography plays a significant role in mediating neurite formation and extension [8, 9]. The development of nanofabrication techniques allows for the fabrication of various types and dimensions of topography on substrate materials [10]. Anisotropic topographies, such as grooves and aligned fibers, have been reported to induce neurite elongation and alignment by providing contact guidance though focal adhesions [11, 12]. Isotropic topographies, such as pillars and holes, influenced cell morphology and cell-cell interaction [13–15].

Cell attachment is necessary for axon growth. Many synthetic biomaterials are not sufficient to regenerate nerves due to the insufficient cell binding. Bioactive molecules, such as extracellular matrix (ECM) proteins and cell adhesive peptides, were immobilized to synthetic materials to improve cells responses [16, 17]. Fucoidan is a fucose-based sulfated polysaccharide, derived mainly from brown algae. Fucoidan has been reported to have versatile bioactivities, such as antioxidant, anti-coagulant, anti-inflammatory, and anticancer activities [18]. A growing number of recent studies have demonstrated that fucoidan exerts a neuroprotective function to treat brain disorders [19, 20]. Fucoidan can block amyloid *β*, thus preventing neural apoptosis [21]. Additionally, fucoidan has been reported to inhibit the mitogen-activated protein kinase signaling pathway and suppress infarct volume in cerebral ischemia-reperfusion injury [19]. Our recently studies showed that fucoidan can substantially increase cell adhesion and bind ECM proteins to improve cellular responses [22, 23]. To this end, we sought to use topography and fucoidan to synergistically improve cell adhesion and neuritogenesis.

PVA is a biocompatible material and has been explored for various biomedical applications [24, 25]. Nerve conduits made of PVA have been approved for clinical use, due to the biocompatibility and tunable mechanical properties for mimicking biological tissues [26–28]. However, PVA nerve conduits failed to promote nerve function recovery for long gap injuries, as PVA is not benign for cell adhesion. Luminal modifications using biochemical and biophysical cues to guide axon growth and improve cell adhesion are required. In our previous studies, PVA was crosslinked by sodium trimetaphosphate (STMP) to generate hydrogels and was able to be fabricated into tubular grafts with varied diameters and tunable mechanical properties [29]. The fabricated PVA hydrogels can be easily patterned with submicron and micron-sized topographies to mediate cell responses, including cell adhesion, proliferation, and migration [22, 30]. Additionally, the hydroxyl groups on PVA allow for chemical reaction to conjugated various biochemical molecules, such as peptide [31, 32], fucoidan [22], and gelation [33], to substantially improve cell adhesion. Thus, in this study, we explore modification strategy of PVA using biochemical molecules and topography to synergistically promote neurite outgrowth and alignment. Micron-sized isotropic topography, 1.8 μm convex (CVX) lenses, and anisotropic topographies, 2 μm and 10 μm gratings were successfully fabricated on PVA hydrogels by soft lithography. Patterned PVA were further conjugated with fucoidan and other ECM proteins. The effects of topography and biochemical molecules on the adhesion, neuritogenesis, and neurite alignment of pheochromocytoma 12 (PC12) cells were evaluated.

## 2. Methods and materials

### 2.1. Fabrication of PVA hydrogels

PVA was crosslinked by STMP (Sigma-Aldrich) as previously reported [30]. Briefly, 10% (w/v) PVA aqueous solution was prepared by dissolving PVA powder (Mw 85 000–124 000, 87%–89% hydrolyzed, Sigma-Aldrich) in deionized (DI) water and autoclaving for 20 min. About 15% (w/v) STMP aqueous solution was prepared freshly. About 2.5 ml of STMP solution was added in 30 g of PVA solution under stirring condition. About 1 ml of 30% (w/v) sodium hydroxide (NaOH; Sigma-Aldrich) was subsequently added dropwise, followed by 5 min stirring to obtain PVA crosslinking solution. The PVA crosslinking solution was subsequently centrifuged for 10 min at 2000 rpm to remove bubbles.

Patterned polydimethylsiloxane (PDMS; SYLGARD 184, Dow Corning) molds with 2 μm gratings (2 μm height, 2 μm line width, and 4 μm pitch), 10 μm gratings (10 μm height, 10 μm linewidth, and 20 μm pitch), and 1.8 μm CVX lenses (1.8 μm diameter, 0.7 μm height, and 2 μm pitch) were used to prepare patterned PVA films, as previously reported [30]. Briefly, PDMS molds were cut into disc shape and loaded in 24 well plates. The PDMS molds were treated with air plasma for 1 min. Immediately after plasma treatment, 700 μl of PVA crosslinking solution was added to each PDMS mold. The plate was centrifuged at 1500 rpm for 1 h. To prepare unpatterned PVA films, crosslinking PVA solution was cast in empty wells of a 24-well plate. After centrifugation, bubbles were gently removed, and the plates were subsequently placed in a wine cooler at 20 °C and 60%–70% humidity for ten days until PVA was fully crosslinked. PVA films was demolded in 10× phosphate-buffered saline (PBS) for 3 h, 1× PBS for 3 h, and DI water overnight. The demolded PVA films were dried in 60 °C oven overnight to remove moisture. Dried unpatterned and patterned PVA films were imaged using an environmental scanning electron microscope (SEM) (FEI Quanta FEG 250 ESEM) in high vacuum mode at 20 kV. Dimension of the fabricated topography was measured using a laser scanning confocal microscope (LEXT OLS4100).

### 2.2. Modification of PVA hydrogels

**Figure 1.**
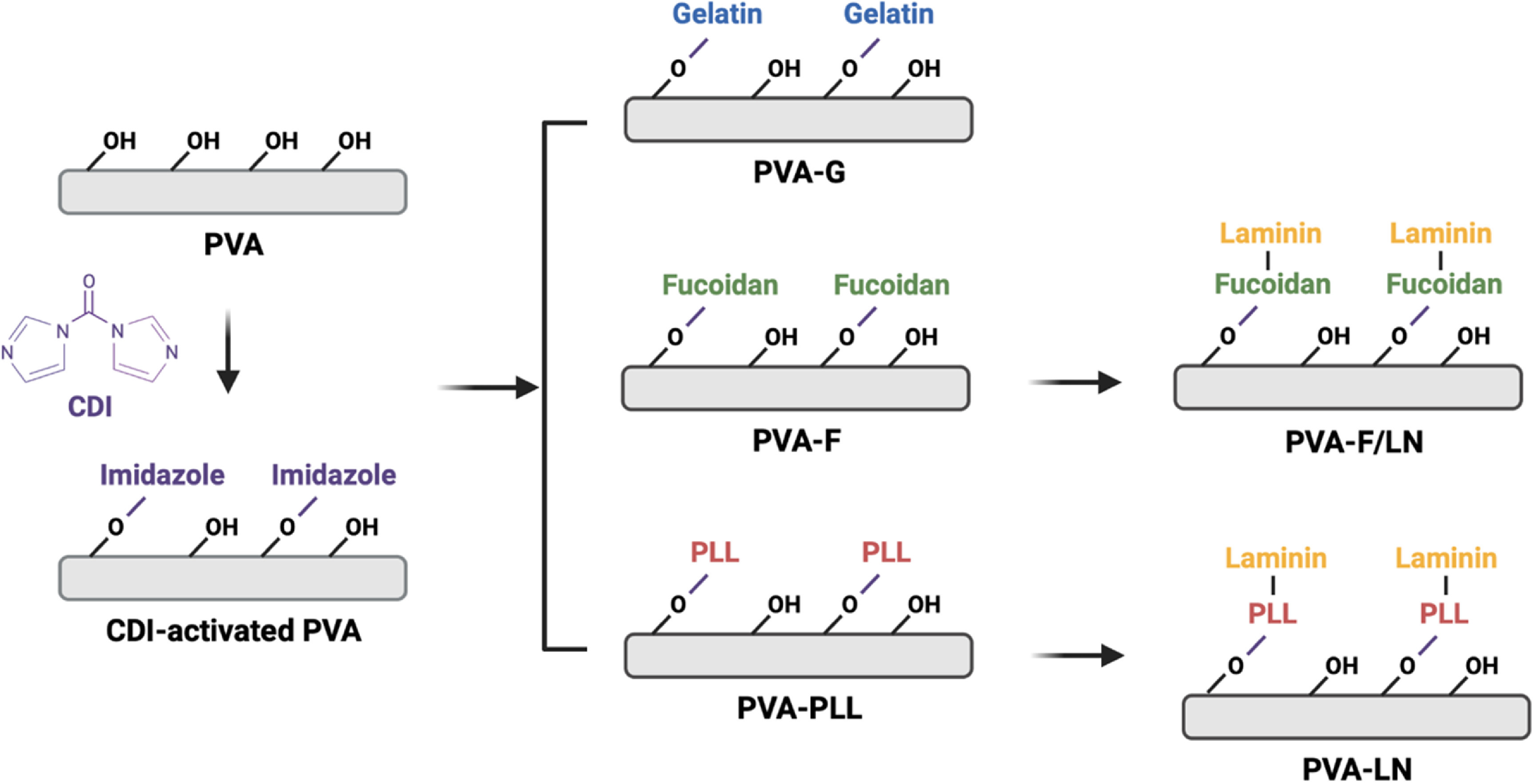
Schematic of gelatin, fucoidan, and laminin modifications on PVA through carbonyldiimidazole (CDI) reaction. PAV-G and PVA-F were prepared by activating PVA with 100 mg ml^−1^ CDI for 1 h at room temperature, followed by overnight incubation in 10 mg ml^−1^ gelatin or fucoidan solution at 37 °C. PVA-LN was prepared by conjugating poly-l-lysine (PLL) to CDI-activated PVA at 4 °C overnight, followed by incubation in 20 μg ml^−1^ laminin for 1 h at 37 °C.

PVA films were modified using carbonyldiimidazole (CDI, Sigma-Aldrich) reaction, as shown in figure 1. Dry PVA films were loaded in a 24-well plate and activated with 500 μl of 100 mg ml^−1^ CDI solution in dimethyl sulfoxide for 1 h at room temperature with 100 rpm shaking. The CDI-activated PVA (PVA-CDI) was washed 3 times in with 1× PBS to remove excess CDI. Gelatin was conjugated to PVA hydrogels by incubating PVA-CDI in 500 μl of 10 mg ml^−1^ gelatin type B (Sigma-Aldrich) solution at 37 °C overnight (PVA-G). The samples were subsequently washed 3 times in 1× PBS to remove excess unconjugated gelatin. To facilitate conjugation, fucoidan (from *Fucus vesiculosus*, Marinova) was aminated as previously reported [22]. Briefly, fucoidan was activated by CDI in formamide (Sigma-Aldrich) for 1 h, followed by adding ethylenediamine (Sigma-Aldrich) in the solution and stir overnight to aminate fucoidan. PVA-CDI was incubated in 500 μl of 10 mg ml^−1^ aminated fucoidan solution at 37 °C overnight to conjugated fucoidan on PVA hydrogels (PVA-F). To modify PVA with laminin (LN), PVA-CDI was first UV-sterilized for 20 min inside a biosafety cabinet and incubated in 500 μl of 0.5 mg ml^−1^ poly-l-lysine (PLL) (Thermo Fisher Scientific) solution at 4 °C overnight (PVA-PLL). The PVA-PLL samples were sterilized using antibiotics as described below. Sterilized PVA-PLL was subsequently incubated with 500 μl of 20 μg ml^−1^ LN (mouse LN, Grand Island Biological Company) solution at 37 °C for 1 h and washed with sterile 1× PBS once for 5 min (PVA-LN).

### 2.3. Fabrication of PVA conduits

PVA small diameter conduits were fabricated as previously described [22]. Briefly, PDMS films with 2 μm gratings were attached to the surface of a metal wire. The metal wire was treated with air plasma for 1 min, immediately immersed into PVA crosslinking solution, and sonicated for 1 h. Afterwards, PVA crosslinking solution was dip-coated on the cylinder rod to form conduits. The PVA hydrogel conduits were fully crosslinked for three days in a wine cooler at 20 °C and 60%–70% humidity and demolded using PBS. The PVA conduits with 2 μm gratings were subsequently modified with fucoidan as described above.

To visualize the quality of luminal structure, the PVA conduits were cut open to expose the lumen and imaged using a laser scanning confocal microscope (LEXT OLS4100).

### 2.4. Sterilization of PVA hydrogels

To sterilize PVA samples for cellular experiments, unmodified and modified PVA samples were incubated in 500 μl of antibiotic solution consisting of 1% amphotericin B and 1% penicillin-streptomycin (PS) at 4 °C overnight, followed by 20 min of UV sterilization inside a biosafety cabinet. Antibiotic solution was aspirated, and autoclaved Teflon rings were inserted into each well to hold the PVA samples down. PVA samples were incubated in fresh antibiotic solution at 37 °C for another 30 min and washed 5 times in 1× PBS. The sterilized samples were directly used for cell seeding or for studying ECM and growth factor coating in the next section.

### 2.5. LN and nerve growth factor (NGF) coating

To coat samples with LN, PVA, PVA-G, and PVA-F were incubated in 500 μl of 20 μg ml^−1^ LN solution at 37 °C for 1 h and washed with sterile 1× PBS once for 5 min before cell seeding. To compare the amount of LN on LN-coated PVA, PVA-G, and PVA-F, 5% of Alexa 488 fluorescent-labeled LN was mixed with non-fluorescent LN. The LN-coated samples were imaged with Zeiss fluorescence microscope (Axio Observer Z1), and the fluorescence intensity was analyzed using Fiji software.

To bind NGF, sterile PVA, PVA-G, and PVA-F were incubated in 100 μl of 100 μg ml^−1^ recombinant human beta-NGF (*β*-NGF, PeproTech, Inc.) solution at 37 °C for 1 h and used directly for cell seeding. To compare the coated NGF on PVA, PVA-G, and PVA-F, the NGF-coated samples were stained with NGF antibody (PeproTech, Inc.) and imaged with Zeiss fluorescence microscope (Axio Observer Z1). The fluorescence intensity was analyzed using Fiji software.

### 2.6. Maintenance of PC12 cells

PC12 cell lines from American Type Culture Collection were cultured with Dulbecco’s Modified Eagle’s Medium (DMEM)-high glucose with 4.5 g l^−1^ glucose, L-glutamine, and sodium bicarbonate (Sigma-Aldrich), 10% horse serum (Grand Island Biological Company), 10% fetal bovine serum (FBS) (Grand Island Biological Company), and 1% PS (Grand Island Biological Company). The specific volume for each substance is shown below in table 1.

**Table 1.** Composition of maintenance media for PC12 cell culture.

Reagent	Volume	Final concentration
DMEM (high glucose)	39.5 ml	—
Horse serum	5 ml	10%
Fetal bovine serum (FBS)	5 ml	10%
Penicillin streptomycin (10 000 μg ml^−1^)	0.5 ml	1%

PC12 cells were cultured in collagen IV-coated 6-well plates or T-25 cm cell culture flasks. Briefly, 6-well plates or T-25 cm cell culture flasks were coated with collagen type IV from human placenta (Sigma-Aldrich) at a coating density of 10 μg cm^−2^ at 37 °C for at least 6 h or at 4 °C overnight. Excess collagen solution was removed, and the surfaces were allowed to dry in an incubator at 37 °C. Before seeding the cells, the plates/flasks were washed with 1× PBS three times for 5 min each time. PC12 cells were seeded at a density of 50 000 cells cm^−2^ and cultured in maintenance media in an incubator at 37 °C with 5% CO_2_ and 99% humidity. About 50% of the maintenance media was changed every other day. After the cells reaching 80% confluency, the supernatant culture media was aspirated. About 3 ml fresh maintenance media was added back to each well of the 6-well plate or 7 ml for the T-25 cell culture flask. A 1 ml pipette tip was used to gently scrape the cells in different directions to detach the cells from the plate surface. The cell suspensions were transferred to a 15 ml centrifuge tube and pipetted to dissociation cell aggregates into the single cell suspension before performing cell counting with a hemocytometer.

### 2.7. Induction of PC12 neurite outgrowth

Neurite outgrowth of PC12 cells was induced by differentiation media composed of DMEM-high glucose medium, 5% FBS, 1% 10 000 μg ml^−1^ PS, recombinant human *β*-NGF in DI water with 0.1% bovine serum albumin (Sigma Aldrich) and 1 μg ml^−1^ LN mouse protein (Grand Island Biological Company), as shown in table 2.

**Table 2.** Composition of differentiation media for PC12 neurite outgrowth.

Reagent	Volume	Final concentration
DMEM (high glucose)	44.5 ml	—
Fetal bovine serum	5 ml	5%
*β*-Nerve growth factor (100 μg ml^−1^)	15 μl	30 ng ml^−1^
Mouse laminin	50 μl	1 μg ml^−1^
Penicillin streptomycin (10 000 μg ml^−1^)	0.5 ml	1%

### 2.8. PC12 viability and proliferation on PVA hydrogels

PC12 cells were seeded on PVA hydrogels with maintenance media, as shown in table 1, at a density of 20 000 cells cm^−2^. Cells were maintained for 14 d in an incubator at 37 °C with 5% CO_2_ and 99% humidity. After 14 d, cells were rinsed once with Dulbecco’s phosphate-buffered saline (DPBS) (A1285801 Grand Island Biological Company). Then cells were incubated with 0.25 μl of 4 mM eBioscience Calcein AM Viability Dye (65-0853-39 Invitrogen) and 1 μl of 2 μM ethidium homodimer-1 (E1169 Invitrogen) diluted with DPBS for each well. About 500 μl of the solution was added to each sample and incubated for 30 min at room temperature. Then samples were washed with 1× PBS three times each for 1 min. To stain cells with phalloidin, cells were washed once with PBS following by 15-minute fixation using 4% paraformaldehyde. After fixation, cells were permeabilized with 0.05% Triton X-100 and 50 nM glycine for 20 min and stained with DAPI and Alexa Fluor Phalloidin 546 for 30 min at room temperature. Stained cells were imaged with Zeiss fluorescence microscope (Axio Observer Z1) and analyzed using Fiji.

### 2.9. PC12 neurite outgrowth on PVA hydrogels

PC12 cell were seeded with differentiation media containing NGF and LN at a density of 20 000 cells cm^−2^. Cells were maintained for three days in an incubator at 37 °C with 5% CO_2_ and 99% humidity. After three days, phase-contrast images of cells were taken using Microscope Primovert (415510-1101-000 Carl Zeiss). Viability dye solution was prepared with 0.5 μl of 4 mM calcein-AM, 0.5 μl of penicillin streptomycin and 49 μl of high glucose DMEM. About 50 μl of dye solution was added to each well and the sample was incubated for 15 min at 37 °C. Cells were imaged with Zeiss fluorescence microscope (Axio Observer Z1) and analyzed using Fiji. To determine the percentage of differentiated cells, cell length and neurite length were measured using Fiji, and cells with neurite length that is 1.5 times longer than cell length were considered as differentiated cells [34, 35]. At least 200 cells were counted for each sample group.

## 3. Statistical analysis

All statistical analysis was performed using GraphPad Prism 9. The values of all data were presented as mean ± Standard Deviation (SD). The number of replicas was indicated in the legend of each figure. To determine statistical significance, one-way analysis of variance (ANOVA) with Tukey’s post hoc test was used, as indicated in the figure legends. Statistical significance criterion was set at *p* value of <0.05.

## 4. Results

### 4.1. PC12 adhesion on PVA hydrogels modified with different biochemical molecules

**Figure 2.**
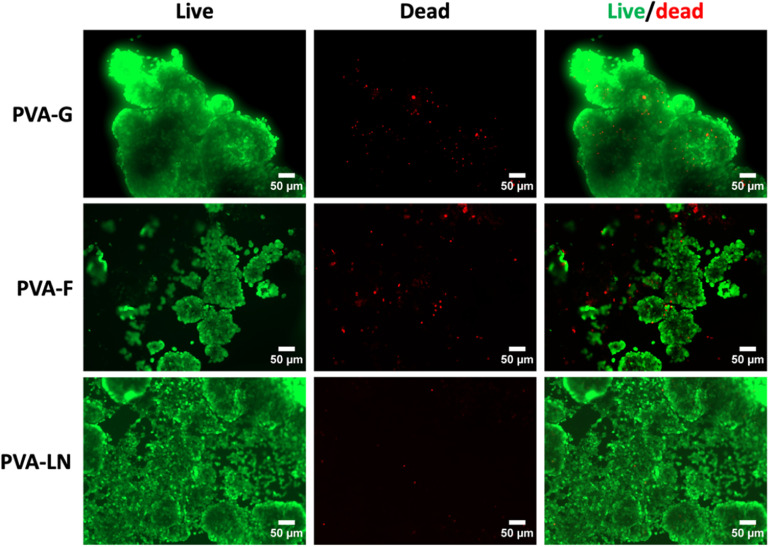
Representative fluorescence microscopy images of the Live/Dead assay of PC12 cell lines on unpatterned PVA hydrogels. PVA surfaces were conjugated with gelatin (PVA-G), fucoidan (PVA-F), and PLL-laminin (PVA-LN). PC12 cell lines were seeded with maintenance media at a seeding density of 20 000 cells cm^−2^. Cells were cultured for 14 d and stained with calcein-AM for live cell (green fluorescence) and ethidium homodimer-1 for dead cells (red fluorescence). The scale bars in all images are 50 μm.

Fucoidan, gelatin and LN were immobilized on PVA hydrogels through CDI reaction, as shown in figure 1. PC12 cell adhesion and viability on PVA-G, PVA-F, and PVA-LN were compared. Viability of PC12 cells was confirmed by live dead staining as shown in figure 2. The majority of PC12 cells remained viable after 14 d culture in maintenance media. PVA-LN supported the most adhesion and cells showed the highest viability with minimal dead cells. The cells on PVA-G and PVA-F also exhibited high viability, suggesting that conjugated gelatin and fucoidan were able to support cell adhesion and growth. However, cells on PVA-G formed aggregates and did not distribute evenly throughout the entire surface, suggesting that cells may have weaker binding to PVA-G compared to PVA-F and PVA-LN.

### 4.2. PVA patterning and characterization

**Figure 3.**
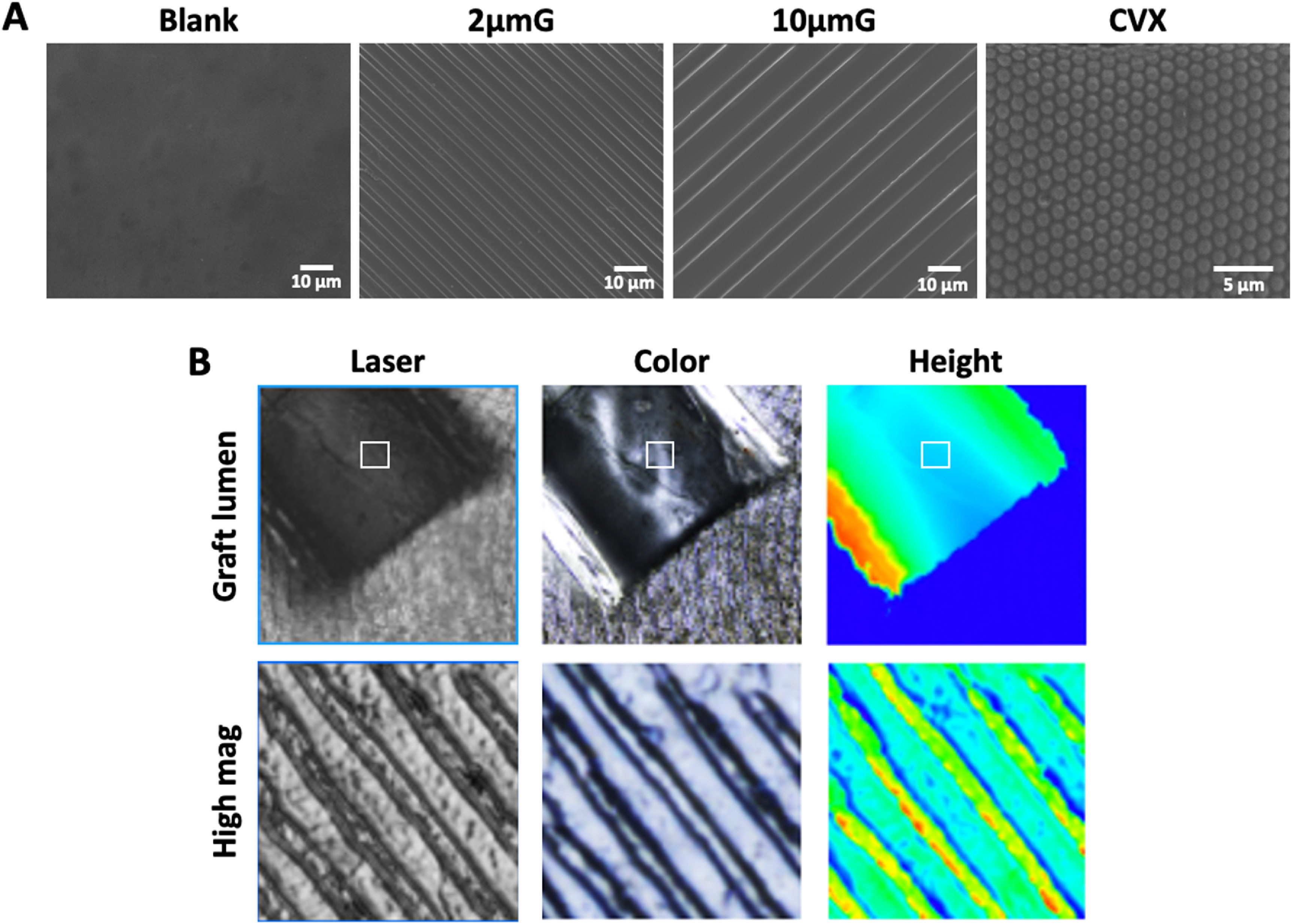
Polyvinyl alcohol (PVA) hydrogel patterning and characterization. (A) Scanning electron microscope (SEM) images of PVA films without pattern (blank) and with 2 μm gratings (2 μmG), 10 μm gratings (10 μmG), 1.8 μm convex lens (CVX). (B) PVA-F tubular grafts with luminal modification of 2 μm gratings (2 μmG). Top panel shows the lumen of PVA-F graft that was cut open longitudinally. Bottom panel shows the 2 μmG topography on the luminal surface of PVA-F grafts.

PVA films with 3 topographies, 2 μm grating (2 μmG), 10 μm grating (10 μmG) and 1.8 μm CVX lens, were fabricated. SEM images verified the successful fabrication of the three topographies on PVA hydrogels (figure 3(A)). The cross-sections of the patterned PVA samples were visualized, and the profiling measurement showed that the fabricated topographies had dimensions close to theoretical dimensions (supplementary figure 1).

To demonstrate the capability of fabricating PVA nerve grafts with luminal patterns. We fabricated PVA-F tubular grafts with 2 μmG luminal topography. The graft was cut longitudinally to expose the luminal surface and imaged with a 3D measuring laser microscope, as shown in figure 3(B). High magnification images proved the presence of 2 μm gratings on the luminal surfaces, and the cross-sectional measurement showed a good fidelity of the topography (supplementary figure 2).

### 4.3. Neuritogenesis of PC12 cells in media with different compositions

PC12 cell culture media with different compositions, as listed in table 3, were compared to determine the optimal media composition to induce PC12 neurite outgrowth. PC12 cells were seeded on PVA-F with basal media (Media B), basal media with 30 ng ml^−1^ NGF (Media D), basal media with 30 ng ml^−1^ NGF, supplemented with 2% or 5% FBS (Media D-2 or Media D-5), or basal media with 30 ng ml^−1^ NGF, supplemented with 5% FBS and 1 μg ml^−1^ LN (Media D-5/LN).

**Table 3.** Composition of media used in inducing PC12 neurite outgrowth.

Name	Composition
Media B	DMEM + 1% Penicillin streptomycin (PS)
Media D	DMEM + 1% PS + 30 ng ml^−1^ NGF
Media D-2	DMEM + 1% PS + 30 ng ml^−1^ NGF + 2% FBS
Media D-5	DMEM + 1% PS + 30 ng ml^−1^ NGF + 5% FBS
Media D-5/LN	DMEM + 1% PS + 30 ng ml^−1^ NGF + 5% FBS + 1 μg ml^−1^ laminin

**Figure 4.**
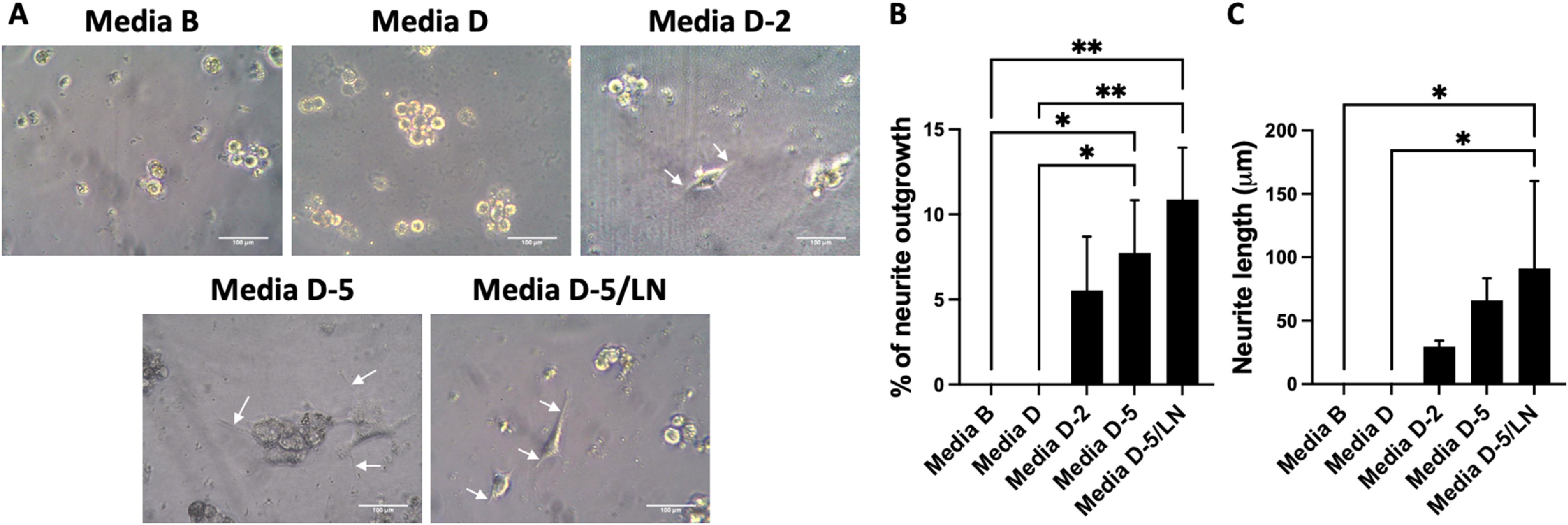
Neurite outgrowth of PC12 cells cultured with various compositions of media. (A) Representative phase-contrast images of PC12 cells cultured with various compositions of media. The PVA surfaces were conjugated with fucoidan (PVA-F) and the seeding density of PC12 was 20 000 cells cm^−2^. Scale bar = 100 μm. White arrows indicate neurite formation. (B) Percentage of PC12 cells that extended neurites. (C) Average neurite length. *n* = 3, * and ** indicates a significant difference using one-way ANOVA *p* < 0.05 and *p* < 0.01, respectively.

After cultured in Media B for three days, the PC12 cells on PVA-F did not show any sign of neurite formation. In media containing 30 ng ml^−1^ NGF (Media D), the number of adhered cells increased; however, the NGF in media D did not induce PC12 cell differentiation (figure 4(A)). With the addition of 2% FBS in culture media, the PC12 cells began to form neurites, and the percentage of cells with neurite outgrowth was further increased as the concentration of FBS in media increased from 2% (Media D-2) to 5% (Media D-5). Adding 1 μg ml^−1^ LN in Media D5 (Media D-5/LN) further promoted the PC12 neurite outgrowth and neurite length of PC12 cultured in Media D-5/LN was the longest compared the cells cultured in other media (figures 4(B) and (C)).

### 4.4. Neuritogenesis of PC12 on PVA modified with biochemical molecules and topographies

**Figure 5.**
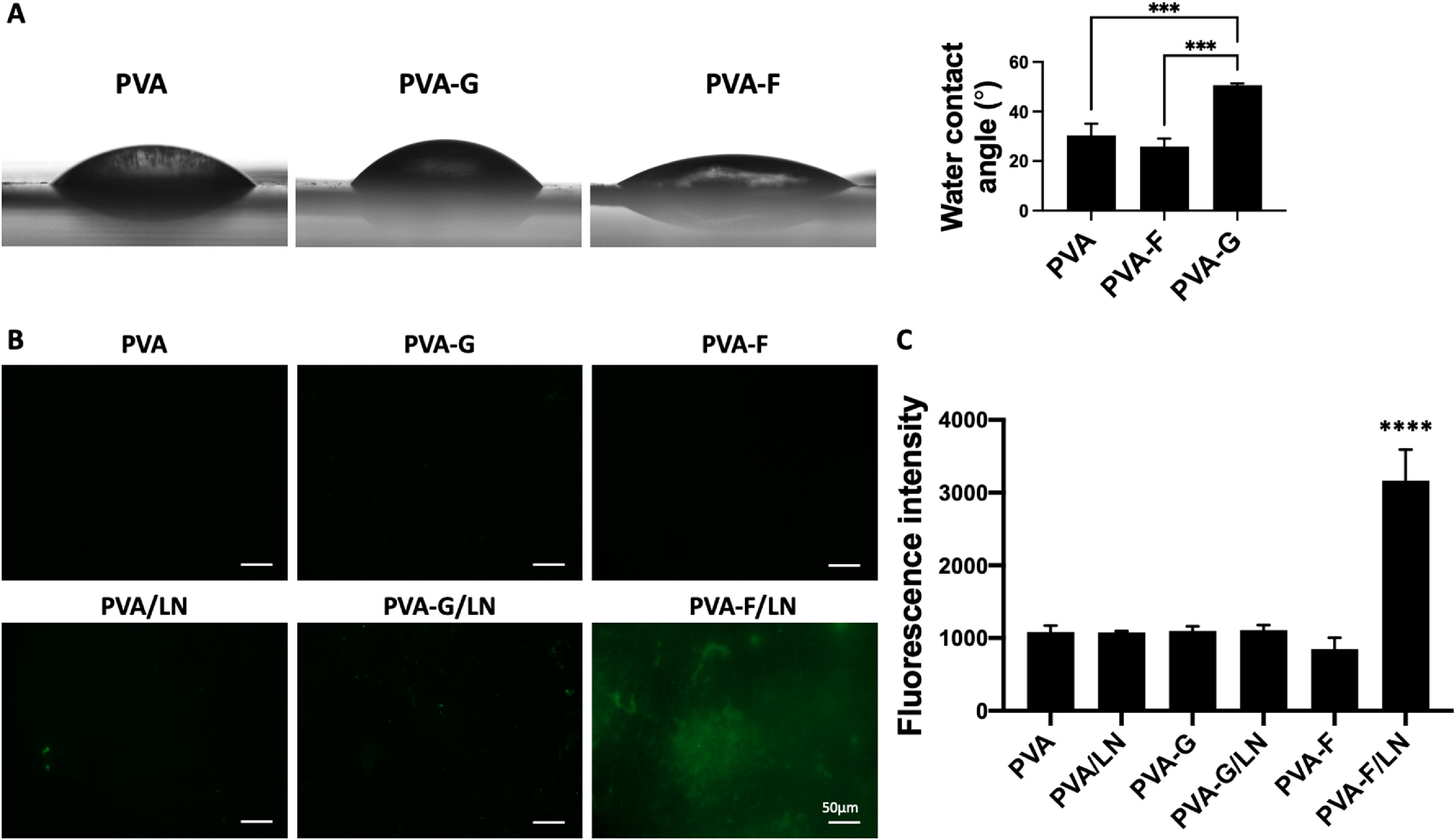
Laminin coating on PVA, PVA-F, and PVA-G hydrogels, (A) Contact angle measurement of PVA, PVA-F, PVA-G hydrogels. *n* = 3, *** indicates a significant difference using one-way ANOVA *p* < 0.001. (B) Representative fluorescence images of LN-coated PVA hydrogels. The surfaces were modified with Gelatin (PVA-G) and fucoidan (PVA-F), and LN-coated PVA-G (PVA-G/LN) and LN-coated PVA-F (PVA-F/LN). PVA and LN-coated PVA (PVA/LN) were used as controls. Green denotes laminin staining. (C) Quantification of fluorescence intensity from laminin staining. *n* = 3, **** indicates a significant difference using one-way ANOVA *p* < 0.0001.

In our previous study, PVA-F has shown capability to present ECM protein, fibronectin, on the surface and improve cell adhesion [22]. To investigate if LN can be presented by PVA-F, we performed LN coating on PVA, PVA-G, and PVA-F and compared LN density on the surfaces. Surface wettability affects the protein adsorption. Prior to LN coating, we performed water contact angle measurement on PVA, PVA-G, and PVA-F. PVA-G exhibited significantly higher contact angle compared to PVA and PVA-F, while the contact angle values of PVA and PVA-F were comparable (figure 5(A)). The LN density on the surface of PVA, PVA-G, and PVA-F was compared by staining the samples against LN antibody (figure 5(B)). PVA-F had significantly higher fluorescence intensity (*p* < 0.0001), indicating that PVA-F was able to present more LN on the surface than PVA and PVA-G (figure 5(C)). Thus, LN-coated PVA-F (PVA-F/LN) was included in the study of PC12 cell differentiation.

**Figure 6.**
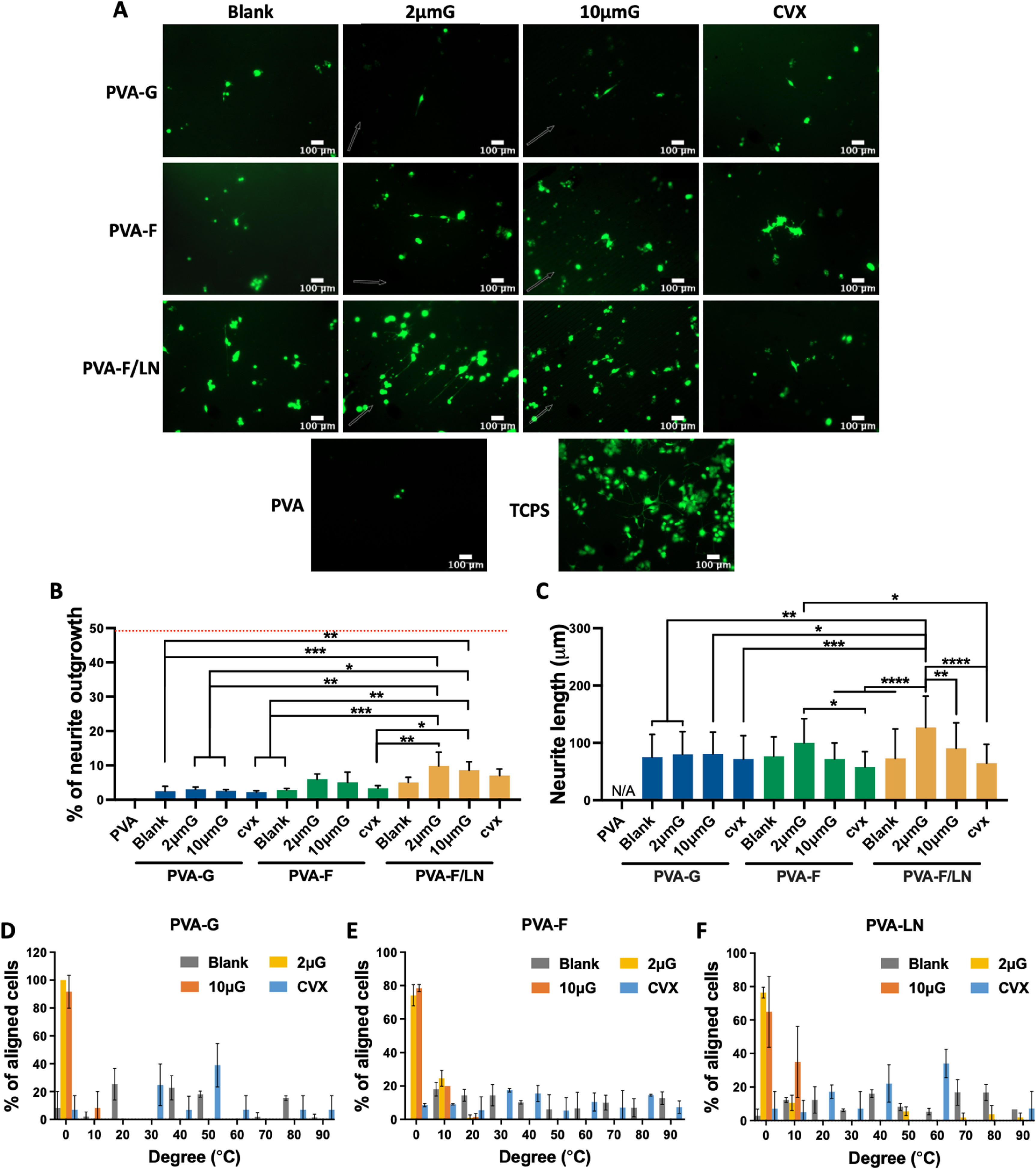
Neurite outgrowth and alignment of PC12 on PVA hydrogels with various topographical and biochemical modifications. (A) Representative fluorescence images of PC12 cell lines. PVA surfaces were modified with fucoidan, gelatin and fucoidan coated with laminin (LN) on different topographies: blank, 2 μG, 10 μG, and CVX. Unmodified PVA and collagen-coated TCPS were used as negative and positive controls, respectively. Cells were seeded at 20 000 cells cm^−2^ and cultured in media D-5 with laminin (media D-5/LN) for three days. PC12 cell lines were Live/dead stained after culturing for three days. The arrows indicate the direction of gratings. The scale bars in all images are 100 μm. (B) Percentage of PC12 cells that extended neurites and (C) average neurite length. *N* = 3, *, **, ***, and **** indicate a significant difference using one-way ANOVA with *p* < 0.05, *p* < 0.01, *p* < 0.001, and *p* < 0.0001, respectively. Neurite alignment of PC12 on PVA-G (D), PVA-F (E), and PVA-LN (F) with different topographies. About 0° indicates that neurites were aligned in the reference direction, and 90° indicates that neurites were perpendicular to the reference direction. For 2 μG and 10 μG samples, grating axis was selected as reference direction, while for blank and CVX samples, a random direction was selected as reference direction.

To study the synergistic effect of topography and biochemical modifications on PC12 neurite outgrowth, we modified both blank and patterned PVA with gelatin, fucoidan, and fucoidan coated with LN. To confirm the adhesion of cells, PC12 cells were culture on PVA-F/LN in growth media for three days and stained with phalloidin, and cells on collagen-coated tissue culture polystyrene (TCPS) were included as a positive control. As shown in supplementary figure 3, cells adhered to LN coated PVA-F (PVA-F/LN) and collagen-coated TCPS, but remained spherical, without the induction of neurite outgrowth. Calcein AM staining showed similar cell morphology and cell density, suggesting that calcein AM could be used to visualize adhered cells. Thus, to better visualize the neurites and avoid neurite retraction due to multiple washing steps in phalloidin staining, cells were cultured for three days in media D-5/LN and stained with calcein AM for assessing neurite outgrowth (figure 6(A)). Few cells attached on PVA, and no neurite extension was observed. Conjugated gelatin and fucoidan on PVA substrates improved PC12 cell adhesion, and induced neurite extension. PVA-F/LN further promoted cell adhesion and neurite outgrowth. On 2 μm and 10 μm gratings, more cells formed neurites and longer neurites were observed compared to blank surfaces and CVX lenses, and the neurite extensions were aligned along the grating axis.

Percentage of PC12 cells that extended neurites were quantified and shown in figure 6(B). About 2.2%–3.1% PC12 cells on PVA-G had neurite outgrowth, and the topographical modification did not alter the percentage of cells with neurite outgrowth (*p* > 0.999). Blank and CVX-patterned PVA-F showed similar level of PC12 neurite formation; while the addition of 2 μm gratings and 10 μm gratings increased the percentage of PC12 with neurite outgrowth from 2.8 ± 0.5% to 6.0 ± 1.5% and 5.1 ± 3.1%, respectively. More PC12 cells on blank PVA-F/LN extended neurites compared to those on blank PVA-G and PVA-F. Moreover, the topographical patterning of PVA-F/LN with 2 μm gratings and 10 μm gratings enabled 9.9 ± 4.0% and 8.6 ± 2.5% of PC12 to form neurite outgrowth, respectively, which are significantly higher than all PVA-G groups and blank and CVX-patterned PVA-F groups.

To characterize the neurite extension, the average length of neurites formed by PC12 cells on each sample was measured. As shown in figure 6(C), cells on PVA-G had comparable neurite length among all groups. On the contrary, in the PVA-F and PVA-F/LN groups, 2 μm gratings enabled longer neurite formation. The average neurite length of PC12 cells on PVA-F with 2 μm gratings was 100.1 ± 42.0 μm, significantly higher than that of cells on PVA-F patterned with CVX lenses (57.6 ± 27.2 μm) and PVA-F/LN patterned with CVX lenses (64.7 ± 32.9 μm). Cells on PVA-F/LN with 2 μm gratings formed the longest neurites (126.8 ± 54.5 μm), significantly longer than cells on all PVA-G samples, PVA-F patterned with 2 μm gratings and CVX lens, blank PVA-F/LN, and PVA-F/LN with other patterns. In addition, neurite orientation on different topographies was measured (figures 6(D)–(F)). Cells on grating structures extended their neurites mainly along the grating axis; while cells on blank and CVX formed neurites in random directions.

### 4.5. Neuritogenesis of PC12 on NGF-coated PVA substrates

**Figure 7.**
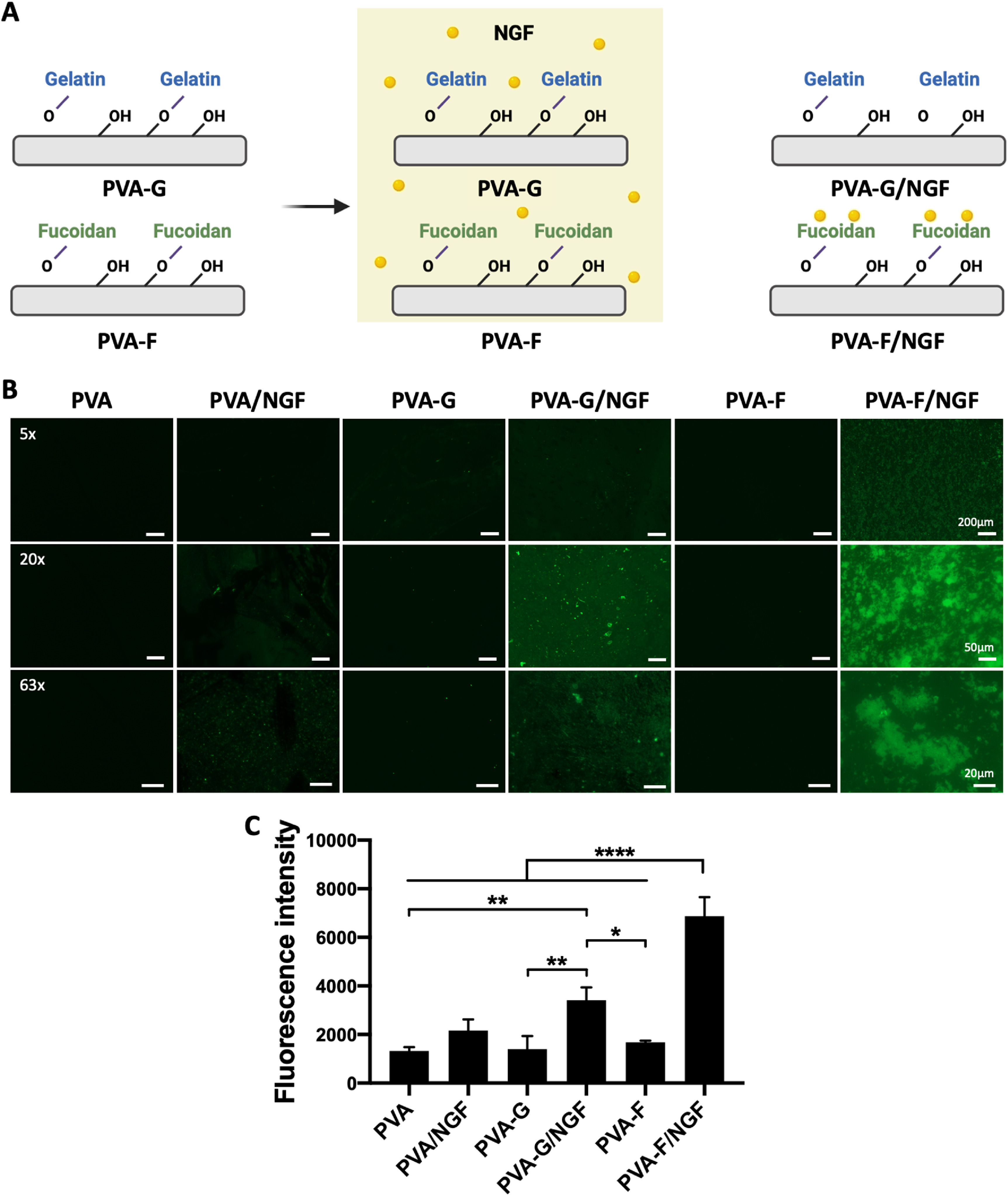
Characterization of nerve growth factor (NGF)-coated PVA hydrogels. (A) Schematic diagram of nerve growth factor (NGF) coating on PVA-G and PVA-F. (B) Representative fluorescence images of NGF-stained surfaces. The surfaces were modified with Gelatin (PVA-G) and fucoidan (PVA-F), and NGF-coated PVA-G (PVA-G/NGF) and NGF-coated PVA-F (PVA-F/NGF). PVA and NGF-coated PVA (PVA/NGF) were used as controls. (C) Quantification of fluorescence intensity. *n* = 3, *, **, and **** indicates a significant difference using one-way ANOVA *p* < 0.05, *p* < 0.01, and *p* < 0.0001, respectively.

NGF has been shown to be essential for PC12 cell differentiation [36]. To examine if NGF can be presented by PVA and modified PVA hydrogels, we coated PVA, PVA-G, and PVA-F with recombinant human *β*-NGF, as shown in figure 7(A). After coating, PVA-F had significantly higher concentration of NGF present on the surface compared to PVA alone and PVA-G, as shown in figures 7(B) and (C).

**Figure 8.**
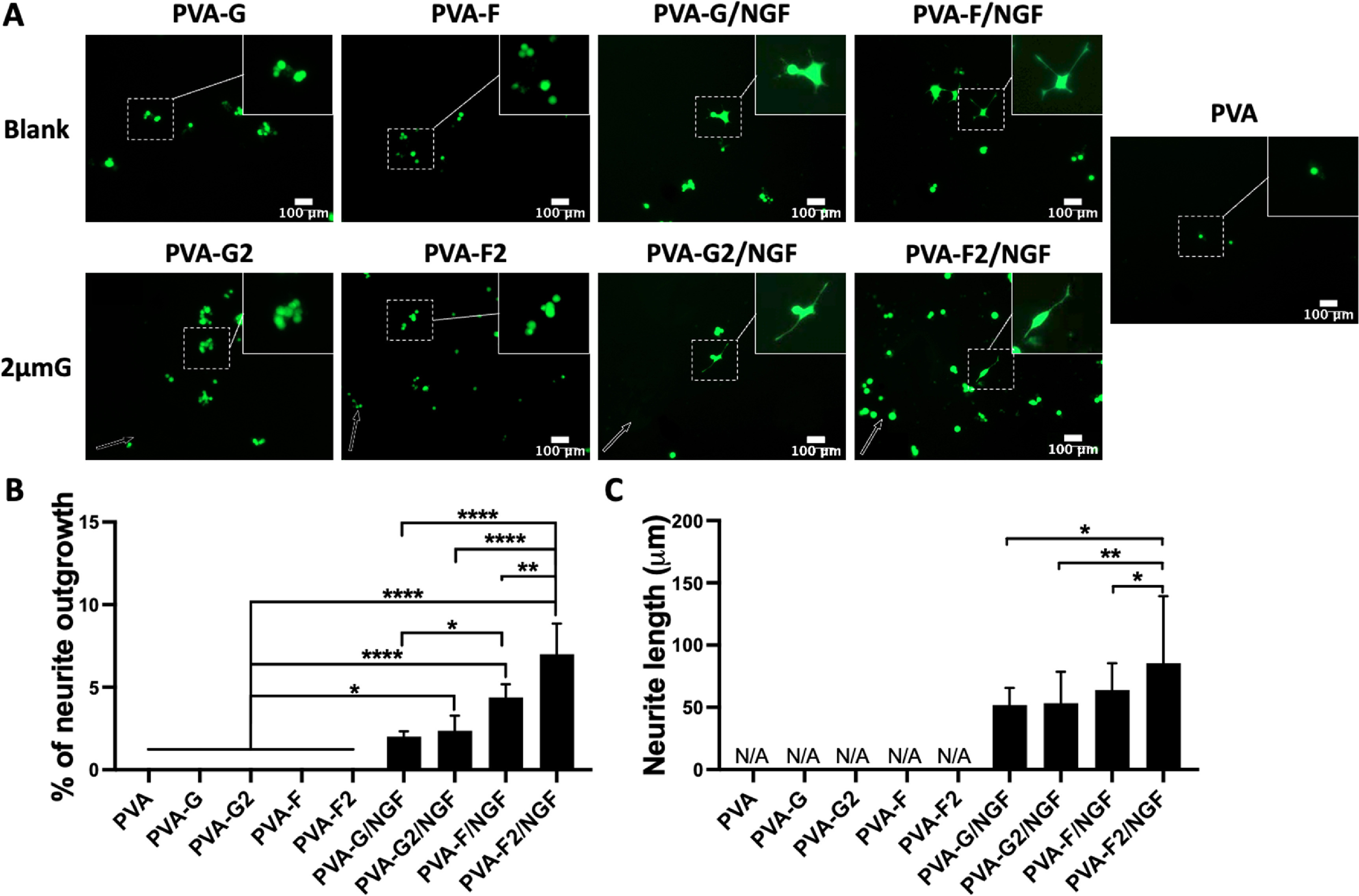
Neuritogenesis of PC12 on nerve growth factor (NGF)-coated PVA hydrogels. (A) Representative fluorescence images of PC12 cell lines. Cells were cultured with media composed of DMEM, 1% PS and 5% FBS and seeded on both blank and 2 μm gratings (2 μmG) substrate. The surfaces were modified with gelatin (PVA-G) and fucoidan (PVA-F), and NGF-coated PVA-G (PVA-G/NGF) and NGF-coated PVA-F (PVA-F/NGF). (B) Percentage of PC12 cells that extended neurites and (C) average neurite length. *n* = 3, *, **, and **** indicate a significant difference using one-way ANOVA with *p* < 0.05, *p* < 0.001, and *p* < 0.0001, respectively.

To explore the potential of NGF coated PVA hydrogels in inducing PC12 cells to extended neurites, we cultured PC12 cells in NGF- and LN-free media for three days and stained with calcein AM. Cells did not show sign of neuritogenesis on PVA, PVA-G, and PVA-F samples with the absence of NGF coating, as shown in figure 8(A). With NGF coating, PC12 cell formed neurites on both blank and 2 μm grating-patterned samples. Neurites formed by PC12 cells on 2 μm gratings extended along the grating axis, while cells on blank surfaces extended their neurites in random directions. The percentage of cells with neurite outgrowth and the average neurite length were further measured and shown in figures 8(B) and (C). Without NGF coating, PC12 cells has 0% of neuritogenesis. About 2.0 ± 0.3% cells formed neurites on blank PVA-G/NGF has differentiation, and PVA-G/NGF with 2 μm gratings (PVA/G2/NGF) increased the percentage to 2.4 ± 0.9%. PC12 cells on blank PVA-F/NGF had 4.4 ± 0.8% neurite outgrowth, significantly higher than that on blank PVA-G/NGF. PVA-F/NGF with 2 μm gratings (PVA-F2/NGF) induced the highest percentage of PC12 neuritogenesis (7.0 ± 1.8%), demonstrating a significantly increase compared to the rest of the groups. The results suggested that fucoidan could present NGF on the surface and enable PC12 neurite outgrowth even without the presence of NGF.

## 5. Discussion

Neurological diseases are causing significant burden to the clinics and cause high rate of mortality and morbidity. Peripheral nervous system has been regarded as self-regenerative compared to central nervous system. However, the regeneration capability of peripheral nervous system is limited by complications such as apoptosis of neurons, inflammation, and scar tissue formation. When nerve defect is longer than 3 cm, nerve grafting is usually required. In the past decades, artificial nerve grafts have been explored and developed to bridge long nerve gaps and support axon growth; however, many have reported that the artificial grafts do not have comparable regeneration levels to autologous nerve grafts, due to the lack of proper biochemical and biophysical cues for axon growth [5, 8]. Thus, in this study, we aim to develop a platform with precisely designed biochemical and topographical cues to promote and guide neuritogenesis.

PVA is inherently hydrophilic and does not support cell adhesion due to the lack of cell binding sites. Nerve guidance conduits made of PVA has been approve for clinical applications. However, due to low cell binding, PVA failed to effectively connect large nerve gaps. Many efforts have been spent to improve cell adhesion to PVA hydrogels [37]. CDI is a zero length bioconjugation linker that is reactive to nucleophiles, such as amine groups and hydroxyl groups [38]. We previously developed an bioconjugation approach using CDI to immobilize bioactive molecules, such as gelatin [33], RGD-mimetic peptide [32], and fucoidan [22], and effectively improved endothelial cell adhesion on PVA hydrogels. The adhesion of PC12 cells is essential for differentiation. In this study, to improve the adhesion of PC12 cells, we conjugated fucoidan, gelatin, and PLL-LN to PVA through CDI reaction. The conjugated molecules significantly increased the adhesion of PC12 cells in their growth media and maintained good cell viability. LN has been extensively explored for inducing neuronal differentiation and neural regeneration [39]. PLL is commonly used as a co-coating with LN to improve neural cell responses. PC12 cells demonstrated more robust adhesion and enhanced neurite outgrowth on PLL/LN double coating surfaces [40]. In our current study, we conjugated PLL on CDI-activated and PVA, followed by the immobilization of LN. The resulting PVA-LN had higher cell attachment and viability compared to PVA-G and PVA-F, suggesting the potency of LN in promoting PC12 adhesion. Gelatin has been used in neural tissue engineering to mediate axon behaviors and neuronal differentiation [41, 42]. In this study, PC12 cells adhered on PVA-G and maintained viable. Collagen is a major component of ECM. We explored the effect of collagen conjugation on PC12 cell adhesion. We performed collagen conjugation reaction at both neutral pH, 0.02% acetic acid, and 0.05% acetic acid; however, the PC12 cells showed low attachment and the majority of cells dead after 12 d of culture. Collagen has been reported to be more stable and soluble in acidic condition, however, the acidic condition was not favorable for conjugation of amine groups to imidazole carbamate [43]. We speculate that the low attachment of PC12 cells was due to the low efficiency of collagen conjugation on PVA-CDI. Thus, collagen was not included in the PC12 neurite outgrowth study. Fucoidan has been reported to have the potential to act as a potent drug to prevent neuronal cell apoptosis for the intervention of neurodegenerative diseases [44]. In our previous studies, we demonstrated that fucoidan conjugation on PVA could substantially increase endothelial cell adhesion [22, 23]. Here, PC12 cells were able to adhere to PVA-F and maintained similar viability after 12 d of culture compared to PVA-G, suggesting that fucoidan is potent in promoting PC12 cell adhesion and viability.

Followed by the confirmation of PC12 adhesion on biochemically modified PVA, we explored the proper media composition for PC12 neuritogenesis. NGF has been widely reported to be essential for PC12 differentiation [36]. In this study, we found that when cultured on PVA-F, NGF alone is not sufficient to induce PC12 neurite outgrowth. No neurite formation was observed without the presence of FBS, and the neurite outgrowth increased with increasing FBS concentration. Adding of LN further increased neurite extension. We previously demonstrated the conjugated fucoidan could bind to ECM protein, fibronectin, to improve endothelial cell adhesion [22]. In this study, we found that conjugated fucoidan was also able to bind to LN and present LN on the surface and increased the number of adhered cells and cells with neurite outgrowth. PVA alone and PVA-G had low LN present on the surface although PVA-G is significantly less hydrophilic compared to PVA-F, suggesting that the LN on PVA-F was not non-specific adsorption. Further experiments are needed to elucidate the underlying mechanism.

Topography is important biophysical cues to mediate the neuronal responses and interaction with the substrate materials [13]. The neuronal cell responses and neurite directionality were dependent on both the isotropy and the dimension of the substrate topography. Many studies have reported that anisotropic features drive the formation of longer neurites [45]. Comparing the PC12 cells on gratings with those on blank and lens structures, more cells formed neurites on gratings, and the neurite length were significantly higher, regardless of the type of biochemical modifications. Highest percentage of cells with neurite outgrowth and longest neurite formation were found for cells cultured on LN coated PVA-F with 2 μm gratings, suggesting that the LN presented by PVA-F was able to promote PC12 neuritogenesis synergistically with 2 μm gratings. Without LN coating, cells on PVA-F formed more neurites compared to PVA-G, regardless of the substrate topography, suggesting that fucoidan is a potent molecule to promote neuritogenesis. In nerve tissue engineering studies, guiding the neurite extension is one of the main goals. Alignment of cells could drive the neuronal growth and migration, thus benefitting the neuritogenesis. Additionally, alignment provides the correct reconnection of nerve bundles and helps to restore nerve function [46]. Numerous studies have reported that anisotropic topography has more profound guidance on cell neuronal cell and neurite alignment and guidance compared to anisotropic topography [45]. Anisotropic topography preferentially guides axons and enhance nerve regeneration. Grooves accelerates axon regeneration, and axons follow the grooves of the topography [47]. Additionally, PC12 cells were reported to exhibit more aligned neurite outgrowth on 2–3 μm microgrooves compared to those on 10 μm microgrooves [48]. Our results in this paper are in line with previous findings that PC12 on 2 μm gratings are more differentiated and had more extended neurites compared to those on 10 μm gratings. Similarly, in our studies, PC12 cells on the 2 μm gratings and 10 μm gratings aligned along the grating axis, and the neurite outgrowth was in parallel with the grating axis, while PC12 cells on blank and CVX lens topography extended neurites in random directions. Neurite growing direction on grooves is affected by groove width and groove depth [11]. The gratings we used in this study had 1:1 width/height and 1:1 width/gap aspect ratio. Neurites formed on grooves that are shallower patterns lost their orientation, while cells on grooves with the same deep and groove/ridge width remarkably extend their neurites along the groove axis [13, 49]. Our previous studies suggested that increasing the height of 2 μm gratings prevent cell from climbing across the gratings, thus enhancing neurite elongation, alignment and neuronal differentiation. Cells on 2 μm gratings with depth of 2 μm had highest percentage of neuronal differentiation. Computational modeling suggested that cells on topography with height to width aspect ratio of 1 exhibited the most active depth-sensing behavior, as a result of the energetic balance between filopodia adhesion and neurite bending [50]. Taken together, we envision that the 2 μm gratings together with biochemical modifications hold great potential in promoting neuritogenesis.

PC12 is a cell line derived from rat pheochromocytoma. NGF is essential for PC12 growth. In this study, we found that PC12 cells were able to extend neurites in NGF-free media on NGF-coated PVA-F but not PVA nor PVA-G, indicating that conjugated fucoidan was able to bind and present bioactive NGF on the surface. Additionally, the 2 μm gratings on PVA-F/NGF also induced higher percentage of cells with neurite outgrowth and longer neurite formation. Taken together, the results suggested that fucoidan can not only present ECM proteins, but also growth factors.

In our previous studies, PVA hydrogels can be shaped into tubular grafts with tailored diameters and mechanical properties [29, 51]. Intraluminal guidance structure provides support for guiding and regenerating axons. Here, we successfully fabricated PVA tubular grafts with 1.7 mm inner diameter and modified with fucoidan and 2 μm gratings. Our previous studies demonstrated that luminal patterning of PVA tubular grafts with micro-sized gratings promoted cell migration along the grating axis [22]. We envision that the strategies presented in this study will be promising in promoting axon generation and guidance, thus generating more effective synthetic nerve grafts for the repair of peripheral nerve injuries.

## 6. Conclusion

In summary, we have successfully fabricated PVA hydrogels with different topographies and biochemical molecules to mediate PC12 cell adhesion, viability, and neuritogenesis. We used CDI to conjugate gelatin, fucoidan, and LN, and successfully improved PC12 adhesion on PVA hydrogels. Fucoidan could present both ECM protein, LN, and NGF to induce and promote neurite outgrowth of PC12 cells. By combining topography with biochemical modification, we demonstrated that gratings and biochemical cues could synergistically promote neurite formation and guide neurite extension. About 2 μm gratings together with LN-coated PVA-F supported the highest number of neurites and longest neurite extension, and 2 μm gratings with NGF-coated PVA-F were able to guide neurite outgrowth of PC12 cells with the absence of NGF in media. We anticipate that the findings presented in this work is beneficial for development of platform for neuritogenesis, and PVA hydrogels modified with fucoidan and 2 μm gratings hold the promises to serve as nerve grafts.

## Data Availability

All data that support the findings of this study are included within the article (and any supplementary files).
